# Connecting the dots: social networks in the classroom and white matter connections in the brain

**DOI:** 10.1111/jcpp.13647

**Published:** 2022-06-07

**Authors:** Rosa H. Mulder, Mónica López‐Vicente, Andrea P. Cortes Hidalgo, Lisa R. Steenkamp, Berna Güroğlu, Henning Tiemeier, Ryan L. Muetzel

**Affiliations:** ^1^ Department of Child and Adolescent Psychiatry/Psychology, Erasmus MC University Medical Center Rotterdam Rotterdam The Netherlands; ^2^ Generation R Study Group, Erasmus MC University Medical Center Rotterdam Rotterdam The Netherlands; ^3^ Department of Developmental and Educational Psychology, Institute of Psychology Leiden University Leiden The Netherlands; ^4^ Department of Social and Behavioral Sciences Harvard T.H. Chan School of Public Health Boston MA USA

**Keywords:** Bullying behavior, peer victimization, peer acceptance, peer rejection, brain imaging, white matter microstructure, Generation R Study

## Abstract

**Background:**

Peer connections in school classrooms play an important role in social–emotional development and mental health. However, research on the association between children's peer relationships and white matter connections in the brain is scarce. We studied associations between peer relationships in the classroom and white matter structural connectivity in a pediatric population‐based sample.

**Methods:**

Bullying and victimization, as well as rejection and acceptance, were assessed in classrooms in 634 children at age 7. White matter microstructure (fractional anisotropy (FA), mean diffusivity (MD)) was measured with diffusion tensor imaging at age 10. We examined global metrics of white matter microstructure and used Tract‐Based Spatial Statistics (TBSS) for voxel‐wise associations.

**Results:**

Peer victimization was associated with higher global FA and lower global MD and peer rejection was associated with lower global MD; however, these associations did not remain after multiple testing correction. Voxel‐wise TBSS results for peer victimization and rejection were in line with global metrics both in terms of direction and spatial extent of the associations, with associated voxels (*p*_FWE_ <.05) observed throughout the brain (including corpus callosum, corona radiata, sagittal stratum and superior longitudinal fasciculi).

**Conclusions:**

Although based only on cross‐sectional data, the findings could indicate accelerated white matter microstructure maturation in certain brain areas of children who are victimized or rejected more often. However, repeated measurements are essential to unravel this complex interplay of peer connections, maturation and brain development over time.

## Introduction

For school‐aged children, classrooms are the main setting where social bonds outside the family are formed. Relationships with classmates have been related to a variety of developmental outcomes (Rubin, Bukowski, & Laursen, 2011; Wentzel & Muenks, 2016). Bullying involvement is one of the most commonly studied phenomena relating to classroom interactions. Bullying behavior and peer victimization often co‐occur (Veenstra et al., 2005) and both have been related to mental health issues such as depression and even suicide risk (Duan et al., 2020). Another important determinant of mental well‐being of the child in a classroom is the level at which they are accepted or rejected by their peers. Peer rejection can be a form of victimization (Olweus, 1994), yet is different from victimization in that together with peer acceptance they are informative of social status (Wentzel & Muenks, 2016). Peer rejection and acceptance have been associated with factors such as loneliness, self‐esteem, and academic achievement (Kingery, Erdley, & Marshall, 2011; Véronneau, Vitaro, Brendgen, Dishion, & Tremblay, 2010).

Associations between mental health and brain connectivity have been delineated (Muetzel et al., 2018) and brain connectivity is plastic throughout childhood (Lebel & Deoni, 2018). Brain connectivity may be susceptible to social influences around this time, yet research on white matter microstructure and childhood peer relationships has been scarce. White matter microstructure is often described using two commonly derived scalar metrics, fractional anisotropy (FA) and mean diffusivity (MD). In childhood white matter connectivity increases, which is reflected in higher FA and lower MD values (Dean et al., 2015; Giorgio et al., 2010). In earlier research, we showed that higher FA and/or lower MD were found in children with a higher intelligence (Muetzel et al., 2015), fewer behavioral problems (Muetzel et al., 2018), and better sleep (Mulder et al., 2019).

Only a few small studies on white matter microstructure and peer relationships have been performed. One study with 87 8‐to‐13‐year‐olds with traumatic brain injury or orthopedic injuries, showed that severe traumatic brain injury was related to more peer‐nominated peer rejection and victimization, and fewer mutual friendships (Yeates et al., 2013). In addition, more mutual friendships correlated positively to white matter volume in voxels from the posterior cingulum bundle. In a study with 35 females aged between 12 and 30 years, social network size was positively related to FA values in amygdalar connections with left orbitofrontal cortex and left anterior temporal lobe, and negatively to FA in amygdalar connections with the right anterior temporal lobe (Hampton, Unger, Von Der Heide, & Olson, 2016). Importantly, this study focused on the amygdala as the seed region of interest, with many white matter paths still to be explored. Lastly, in study including 63 young adults, higher MD and lower FA in the corpus callosum and corona radiata were found for those that reported more peer verbal abuse in childhood (Teicher, Samson, Sheu, Polcari, & McGreenery, 2010). These results suggest an association between negative peer experiences and white matter development. The sample sizes of these studies were, however, relatively modest, suggesting future work with larger sample sizes may be able to detect more subtle differences while also taking into account additional relevant confounders (e.g., behavioral problems), and improving generalizability.

Here, we therefore investigate associations between social connections and white matter microstructure in a large cohort. We use peer nomination data to ascertain bullying behavior, peer victimization, peer rejection, and peer acceptance, which is a natural and informative way of obtaining data and unique at the scale of the current study. White matter microstructure is studied both on a global and on a whole‐brain voxel‐specific level, to study both spatially homogenous associations with white matter microstructure, and localized associations. As earlier research showed that more favorable child characteristics tend to be related to higher FA and lower MD, we hypothesized for the current study that children who experienced peer victimization, peer rejection or who portrayed bullying behavior will have lower FA and higher MD values. Conversely, we hypothesized that children more accepted by their peers will have higher FA and lower MD values.

## Methods

### Setting

The study was performed in the Generation R Study, a prospective population‐based birth cohort that follows the development of children and their parents from pregnancy onwards. Pregnant women residing in the municipality of Rotterdam, the Netherlands, with an expected delivery date between April 2002 and January 2006 were invited to participate in the study (Kooijman et al., 2016). The Generation R Study is conducted in accordance with the World Medical Association Declaration of Helsinki and has been approved by the Medical Ethics Committee of the Erasmus Medical Center, Rotterdam. Written informed consent was obtained for all participants.

### Study population

Peer nominations were collected at 37 elementary schools, grades 1–2, across 190 classrooms in the Rotterdam area at the age of 7. A total of 4,017 children performed the peer nominations task and provided information on peer relationships with all other children. Of these, 1,590 were participants of the Generation R Study. Of these, 864 children underwent magnetic resonance imaging (MRI) at the age of 10 and 812 children completed a diffusion tensor imaging (DTI) scan. During this time, children are typically still in the same elementary school in the Netherlands. The data of three participants were removed because of incidental findings and 175 were excluded due to insufficient data quality (e.g., failed quality control; Methods S1), leaving a total of 634 children with peer nomination and DTI data available.

### Peer nominations

Peer relationships were assessed in school classes with the PEERS Measure (peer evaluation of relationships at school/in Dutch: *pesten en relaties op school*) (Verlinden et al., 2014), a computerized nomination system. Pictures of all children in a given class were portrayed in the program so that each child could nominate the other children by clicking on their photos. Bullying involvement was assessed with four victimization questions on physical (e.g., pushing or hitting), verbal (e.g., calling names), material (e.g., breaking or hiding belongings), and relational bullying (social exclusion). For each item, children were asked if someone repeatedly portrayed the behavior toward them (maximum of 10 nominations per questions). Bullying behavior was based on the average number of incoming nominations (multi‐informant peer report) on all four questions, adjusted for the total averaged number of possible incoming nominations (meaning class size minus 1). Peer victimization was based on the average number of *out*going nominations (self‐report) on all four questions, adjusted for the total averaged number of possible outgoing nominations (class size minus 1). Peer acceptance and rejection were adjusted incoming nomination scores, measured by asking children who they would or would rather not invite on a school trip, respectively (maximum of 6 nominations per question). The PEERS measure has a good test–retest reliability (average ICC = 0.72), and good internal and external validity (Verlinden et al., 2014).

For a sensitivity analysis (see below), we categorized bullying and victimization using a 75^th^ percentile cut‐off in a mutually exclusive variable (‘uninvolved/victim/bully/bully‐victim’; uninvolved children had a score below the cut‐off for both bullying behavior and peer victimization and bully‐victims had a score above the cut‐off for both) in the full peer nomination sample of *n* = 4,017 (Veenstra et al., 2005).

### MR‐image acquisition

MRI data were acquired on a 3 T General Electric MR750W system (GE, Milwaukee, WI (White et al., 2018)). Diffusion tensor imaging was performed with an echo‐planar imaging sequence (T_R_ = 12,500 ms, T_E_ = 72 ms, Acquisition Matrix = 120 × 120, Field Of View = 240 mm × 240 mm, slice thickness = 2 mm, number of slices = 65). Thirty‐five volumes with diffusion weighing (b = 900 s/mm^2^) and 3 volumes without diffusion weighting (b = 0 s/mm^2^) were acquired.

### MR‐image pre‐ and postprocessing

Images were preprocessed using FSL (Jenkinson, Beckmann, Behrens, Woolrich, & Smith, 2012). Images were adjusted for motion and eddy‐current induced (Jenkinson & Smith, 2001), and the resulting transformation matrices were used to rotate the diffusion gradient direction table (Jones & Cercignani, 2010). Nonbrain tissue was removed (Smith, 2002). The diffusion tensor was fit using robust estimations with the RESTORE (Cook et al., 2006) and common scalar maps (FA, MD, axial diffusivity (AD) and radial diffusivity (RD)) were computed. Automated probabilistic fiber tractography was run using the ‘AutoPtx’ plugin (de Groot et al., 2015). Average DTI scalar metrics were computed for each tract and a global DTI measure for each scalar metric was extracted from confirmatory factor analyses (Table S1) (Muetzel et al., 2015). Whole brain voxel‐based analyses were conducted using Tract‐Based Spatial Statistics (TBSS) (Smith et al., 2006) and ‘Randomize’ (Winkler, Ridgway, Webster, Smith, & Nichols, 2014).

### Statistical analyses

Missing data on covariates (min *n* = 0% for age and sex; max *n* = 14% for household income) were imputed in R version 3.4.3 (R Core Team, 2013) using the *mice* package v3.13.0 (Buuren & Groothuis‐Oudshoorn, 2010) (Methods S2).

The associations between the peer nomination data and white matter microstructure were tested with linear regressions, with peer nomination scores as the predictors and white matter metrics as the outcomes. To mutually adjust peer nomination scores for relevant co‐occurrence, peer victimization and bullying behavior were jointly added as predictors in one model, and peer rejection and peer acceptance in another model. FA and MD were taken as the primary metrics of interest, if associations were found for MD, we followed up with AD and RD analyses. In addition, if associations were found, we ran supplemental individual tract‐based analyses on 12 tracts to complement the TBSS results (Methods S3). To demonstrate the extent and relevance of observed confounding, models were adjusted for covariates in two steps; in the base model, covariates (details in Methods S4) included child sex, age at MRI measurement, diffusion image quality, handedness, and parental national origin, while the fully adjusted model additionally included non‐verbal IQ of the child, maternal education, household income, and child internalizing and externalizing problems. These models were applied in both the analyses with global white matter integrity measures, and in the voxel‐wise TBSS analyses. When estimates did not substantially change from the base to the fully adjusted model, we concluded that estimates are not substantially influenced by observed confounding and presented the results from the fully adjusted model as the main results. *p*‐Values were false discovery rate (FDR) corrected (Benjamini & Hochberg, 1995) across the PEERS measures and global metrics. For each TBSS analysis, a total of 5,000 permutations were run using “Randomize” and significant clusters were identified with a family‐wise error corrected significance threshold of 0.05 (Smith & Nichols, 2009).

### Sensitivity analyses

Several sensitivity analyses were performed. First, as the PEERS measures were positively skewed (Figure S1) but transformation of the measures did not result in normal distributions, results were inspected for possible influential outlier(s). Any of such data points were removed and analyses were repeated (details in Methods S5). Second, children included in the current study more often had parents that were born in the Netherlands, were older, and had mothers with higher education than children in the full Generation R Study. To adjust for potential selection effects, we used inverse probability weighting in a sensitivity analysis (details in Methods S6). Third, given that there are differences in the literature on how bullying involvement is quantified (continuous versus categorical), the global and voxel‐wise analyses were repeated with mutually exclusive categories for bullying and victimization (bully/victim/bully‐victim/uninvolved). Last, to gauge the effect of the mutual adjustment of peer victimization and bullying behavior on the results, and that of peer rejection and peer acceptance, global and voxel‐wise analyses were repeated for each of these four measures individually.

## Results

Sample characteristics are depicted in Table 1. The children were on average 7.3 years old during the PEERS measure and 10.2 years old (*SD* = 0.60) at the time of MRI scanning. The sample consisted of 54.4% girls. Pearson’s correlations among the PEERS measures (Table 2, see Table S2 for a complete correlation matrix) showed that bullying behavior was positively correlated with peer victimization (*r* = .24, *p* < .001) and that peer rejection and peer acceptance were correlated negatively (*r* = −.36, *p* < .001). Bullying behavior was correlated with peer rejection (*r* = .48, *p* < .001). Peer victimization was associated with peer rejection as well (*r* = .14, *p* < .001), but not with peer acceptance (*p* > .05). Bullying behavior and peer acceptance were negatively associated (*r* = −.08, *p* < .05).

**Table 1:** Sample characteristics

*N*	634
Sex, girl (*n* (%))	345 (54.4)
Age at PEERS measure in years (mean (*SD*))	7.28 (0.68)
Number of diffusion‐weighted volumes with signal attenuation (mean (*SD*))	2.15 (1.82)
Age at DTI scan in years (mean (*SD*))	10.23 (0.60)
Handedness, −1 = left, 1 = right (mean (*SD*))	0.72 (0.47)
Parental national origin (*n* (%))	
Dutch	406 (64.0)
Western, non‐Dutch	60 (9.5)
Non‐Western	168 (26.5)
Nonverbal IQ (mean (*SD*))	103.65 (14.29)
Maternal education, university (*n* (%))	193 (30.4)
Household monthly income, euros (*n* (%))	
<2,000	126 (19.9)
2,000–3,200	179 (28.2)
>3,200	329 (51.9)
Internalizing Problems (mean (*SD*))	4.32 (4.30)
Externalizing Problems(mean (*SD*))	3.47 (4.25)
Victimization (mean (*SD*))	0.05 (0.08)
Bullying behavior (mean (*SD*))	0.05 (0.05)
Peer rejection (mean (*SD*))	0.17 (0.14)
Peer acceptance (mean (*SD*))	0.23 (0.13)
Bullying/victimization categorization (*n* (%))	
Uninvolved	430 (67.8)
Victim	93 (15.0)
Bully	74 (11.7)
Bully‐victim	35 (5.5)

**Table 2:** Correlations between peer connection scores

	Peer victimization	Peer rejection	Peer acceptance
Bullying behavior	0.24**	0.48**	−0.08*
Peer victimization		0.14**	0.01
Peer rejection			−0.36**

* *p* < .05.

** *p* < .001.

### Global white matter microstructure differences

As the estimates for the global white matter microstructure analyses did not substantially change from the base model (Table S3) to the fully adjusted model (Table 3; Table S4), here we only report the results from the fully adjusted model. First, peer victimization and bullying behavior were studied together as predictors in association with white matter microstructure measures. Nominally significant associations were found for peer victimization with global FA and MD, with more peer victimization relating to higher FA and lower MD (FA: β = 0.10, 95%CI = 0.02;0.18, *p* = .01; MD: β = −0.09, 95%CI = −0.17;−0.01, *p* = .03, respectively). A follow‐up analysis also showed a negative association between peer victimization and global RD, but no association between peer victimization and AD (Table S5). No association was found between bullying behavior and global FA or MD. Second, peer rejection and peer acceptance were studied together in the same model in association with global white matter microstructure measures. A nominally significant association was found between peer rejection and global MD, with more peer rejection indicating lower MD (β = −0.10, 95%CI = −0.19;−0.01, *p* = .03). A follow‐up analysis also showed a negative association between peer rejection and AD, and no association between peer rejection and RD (Table S5). No association was found between peer rejection and global FA, and no associations were found for peer acceptance with global FA or MD. The associations of peer victimization and rejection with global FA and/or MD in the fully adjusted model did not remain significant after FDR‐correction.

**Table 3:** Associations between PEERS measures and global white matter microstructure

Global metric	Fractional anisotropy				Mean diffusivity			
	β	95% CI	*p*	*p*_FDR_	β	95% CI	*p*	*p*_FDR_
Peer victimization	0.10	0.02; 0.18	.01	.07	−0.09	−0.17; −0.01	.03	.07
Bullying behavior	−0.08	−0.17; 0.01	.06	.12	0.07	−0.02; 0.16	.10	.16
Peer rejection	0.02	−0.07; 0.11	.61	.61	−0.10	−0.19; −0.01	.03	.07
Peer acceptance	0.03	−0.05; 0.11	.42	.48	−0.04	−0.12; 0.04	.39	.48

Results adjusted for sex, age at MRI, image quality, handedness, parental national origin, intelligence, maternal education, household income, internalizing and externalizing problems. FDR, false discovery rate.

There was no evidence of multicollinearity in any of the models. For each model, the mean generalized variance‐inflation factor over all independent variables was GVIF = 1.2, and the maximum was GVIF = 1.6.

### TBSS

**Figure 1:**
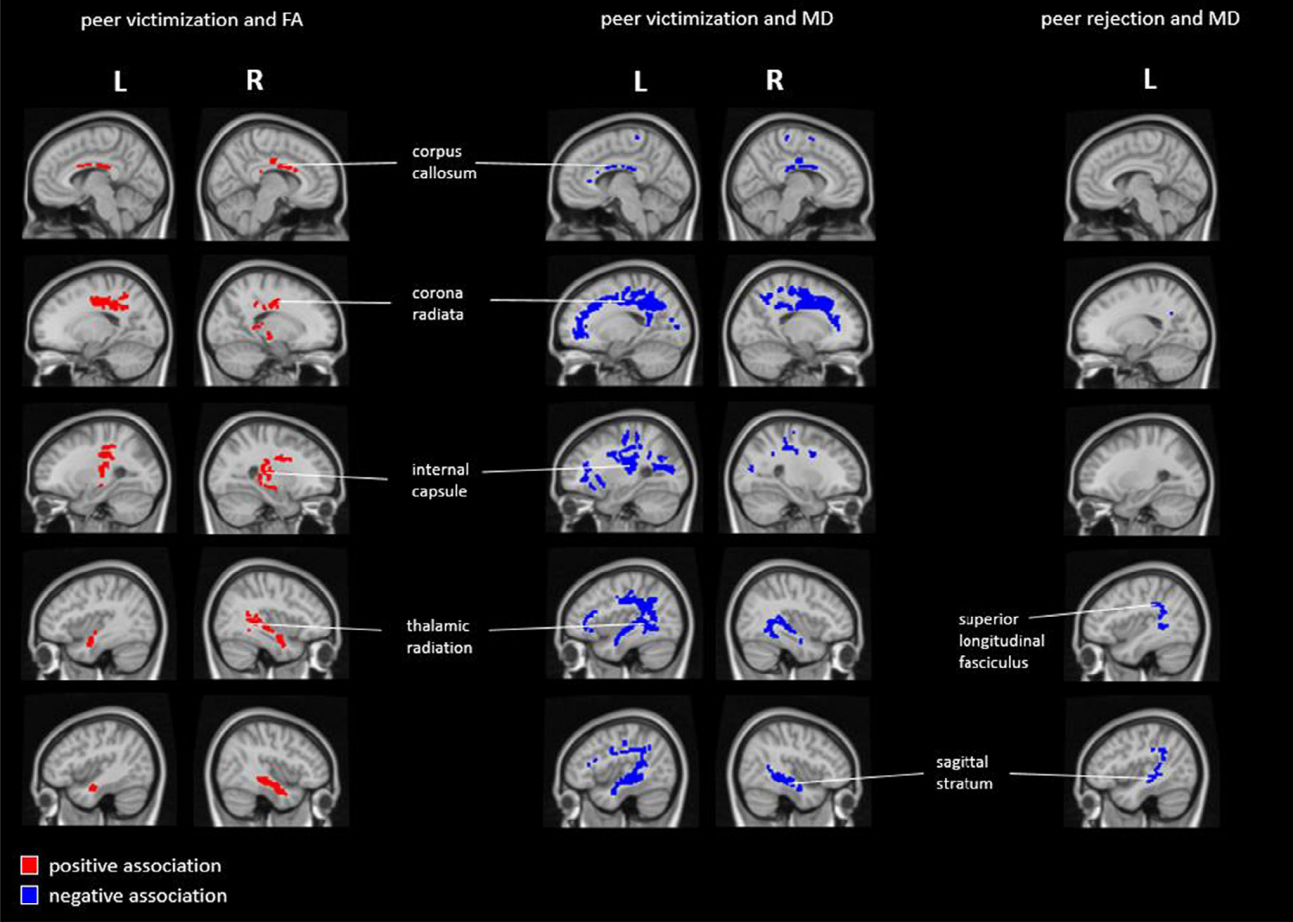
Tract‐Based Spatial Statistics for peer victimization and peer rejection. Blue voxels indicate where peer victimization and peer rejection are negatively associated with MD and red voxels indicate where peer victimization is positively associated with FA. Analyses were adjusted for sex, age at MRI scan, image quality, handedness, parental national origin, nonverbal intelligence, maternal education, household income, and internalizing and externalizing problems, FWE‐corrected [Color figure can be viewed at wileyonlinelibrary.com]

To determine whether there was regional/focal specificity in associations, peer victimization and bullying behavior were jointly examined in association with white matter microstructure in TBSS analyses. We report only TBSS results from the fully adjusted model (*p*_FWE_ <.05), as these are very similar to those for the base model for all the peer nomination scores. Results for peer victimization showed multiple clusters with higher FA, and lower MD for children reporting more peer victimization (Table S6). In short, the largest clusters for FA were found in the corpus callosum, bilateral corona radiata, and the right sagittal stratum. Clusters for MD were more pervasive in the brain, were more often found in the left than right hemisphere (OR = 2.39) and included, among others, the corpus callosum, bilateral corona radiata, bilateral sagittal stratum, and left superior longitudinal fasciculus (Figure 1; Methods S7). In a follow‐up analysis, peer victimization also related to lower RD, but not to AD (Table S7). In tract‐based follow‐up analyses, peer victimization was positively associated (*p*_FDR_ <.05) with FA and negatively associated with MD in the left superior longitudinal fasciculus (Table S8). There were no associations between bullying behavior and FA or MD.

Tract‐Based Spatial Statistics for peer rejection showed no associations with FA. There were multiple clusters with lower MD for children more often rejected by their peers in the base model, yet the number of associated voxels was reduced in the fully adjusted model (Table S9). Associated voxels were more often found in the left than right hemisphere (OR = 68.43) and most were classified to the left sagittal stratum and superior longitudinal fasciculus (Figure 1). A follow‐up analysis did not show any associated voxels for peer rejection with AD or RD in the fully adjusted model. In tract‐based follow‐up analyses, no associations (*p*_FDR_ <.05) between peer rejection and MD were found (Table S10). There were no associations between peer acceptance and FA or MD.

### Sensitivity analyses

#### Influential outliers

After inspection of scatterplots, we identified one outlying data point as potentially influential on the associations of peer victimization with global FA and MD (Figure S3). However, after removal of this point, results remained consistent (Results S1; Table S11; Figures S2 and S4).

#### Selection bias

The results using inverse probability weighting in the global analyses to adjust for potential selection bias effects were consistent with our main results (Table S12). This suggests that findings were robust against selection bias.

#### Categorized PEERS measures

We reran global and TBSS analyses for the fully adjusted model with a categorical victimization and bullying variable: uninvolved/victim/bully/bully‐victim. Associations for victims versus uninvolved with global FA and MD were in similar direction as in our main analyses but did not reach significance (Table S13). No differences in global FA or MD were found between bullies versus uninvolved or bully‐victims versus uninvolved. In the voxel‐wise analyses, we found higher FA and lower MD in victims than in those uninvolved, in line with our main analyses (Table S14). No differences in FA or MD were found between bullies versus uninvolved or bully‐victims versus uninvolved.

#### Individual PEERS measures

We reran the fully adjusted model for each PEERS measure separately and found results for the global to be largely consistent with the main results (Table S15). Voxel‐wise associations were found between peer victimization and FA and MD in similar direction as the main results (Table S16), while those for peer rejection and MD shifted just above the significance threshold. As for the main results, no other associations were found between the PEERS measures and white matter microstructure in the global or voxel‐wise analyses.

## Discussion

We examined associations between peer connections in the classroom with white matter microstructure of the brain at a global and voxel‐based level. For the global white matter analyses, results suggested that children with higher peer victimization scores had marginally higher global FA and lower global MD, and that children with higher peer rejection scores had lower global MD, but these results did not survive correction for multiple testing. Voxel‐wise results were however in line with global results, showing higher FA and lower MD values for higher scores of peer victimization, and lower MD values for higher scores of peer rejection. Associations with peer victimization were found throughout the brain, including the corpus callosum, corona radiata, sagittal stratum, and left superior longitudinal fasciculus, those for peer rejection were more localized in the left superior longitudinal fasciculus. In addition, the associations for MD were more often found in the left rather than right hemisphere. Tract‐based analyses also showed higher FA and lower MD values for peer victimization in the superior longitudinal fasciculus, and associations were more evident in the left rather than the right hemisphere.

The areas in which associations were found facilitate communication among a range of regions. The corpus callosum supports interhemispheric communication and has been related to memory and attention (Wallace, Mathias, & Ward, 2018) but may also be involved in processing of social cues as indicated by differences found for individuals with autism (Aoki, Abe, Nippashi, & Yamasue, 2013), which have also been found in the corona radiata. Further, Muetzel et al. (2019), found in a partly overlapping sample that children who were bullied had increased cortical thickness of the fusiform gyrus—specifically on the left side. This may represent a need for enhanced emotional face processing in the face of threat. The fusiform gyrus is connected to other regions via the sagittal stratum and the superior longitudinal fasciculus. Possibly, regions such as the fusiform gyrus are more innervated for victims of bullying and rejection. Language is another important function for social processing (van den Bedem, Dockrell, van Alphen, Kalicharan, & Rieffe, 2018) and is most often located on the left side of the brain, which might explain the lateralization in results. Additionally, our follow‐up analyses showed that peer victimization was associated with RD and peer rejection with AD, yet directionality was similar for RD and AD in both cases. Earlier research has indicated that the RD metric may be more sensitive to differences in myelination, whereas AD may be more sensitive to fiber density (Winklewski et al., 2018), suggesting that in the current study, both aspects could be involved.

Higher FA and lower MD, AD and RD are associated with neuromaturation and better mental health (Muetzel et al., 2015, 2018). Against this background, the direction of associations for peer victimization and peer rejection was unexpected. We further note that an earlier study reported lower FA and higher MD in relation to peer verbal abuse (Teicher et al., 2010). However, the sample size of the current study was 10 times as large, used concurrent rather than retrospective measurements, and used multi‐informant information. One interpretation of the directionality of these associations is that children who experience peer victimization or peer rejection, have accelerated white matter maturation of various fiber projections. In other words, if a child experiences rejection or victimization, there might be a corresponding acceleration in certain aspects of neurodevelopment. Based on our results, being victimized or rejected by peers could be equated with being 8 months older in global white matter microstructure (based on the estimated age coefficients in the same model). The psychosocial acceleration theory posits that cues of adversity may trigger the body to accelerate development toward puberty in order to enhance reproductive options (Belsky, 2012). Family conflict has for example been related to younger age of menarche in girls (Moffitt, Caspi, Belsky, & Silva, 1992). In a recent region‐of‐interest MRI study on over 2,000 children, it was shown that a stressful family environment was associated with higher FA in the anterior cingulate cortex and that this association was partially mediated by advanced pubertal stage (Thijssen, Collins, & Luciana, 2020). On the other hand, in a meta‐analysis of child maltreatment and voxel‐based white matter microstructure (Lim, Howells, Radua, & Rubia, 2020), reductions in FA were found for maltreated children in several areas, thereby questioning the psychosocial acceleration theory. However, many of the studies used retrospective reporting and had exposed groups smaller than *n* = 20. Therefore, more research is needed to understand the intricacies of early life adversity in the context of white matter maturation. Specifically, understanding the timing of effects is crucial in the interpretation of these findings. While the 3‐year stability of peer victimization in children in this age range was estimated to be moderate (Pouwels, Souren, Lansu, & Cillessen, 2016) and switching between schools is relatively infrequent for Dutch children in this age range, it may be that this or other relevant changes to the social environment took place in between the PEERS and MRI assessments. Additionally, while the PEERS measurement took place 3 years before MRI, we cannot exclude the possibility that similar variations in white matter microstructure already were present before peer victimization and rejection took place. Previous findings (Yeates et al., 2013) that children with severe traumatic brain injury are more often victimized and rejected by peers, indicate a very clear example that white matter differences might precede negative peer experiences. Hence, these differences in white matter microstructure may be indicators of children at risk of negative peer experiences. A repeated measures design could elucidate the directionality of the associations reported here.

Although higher FA and lower MD might be related to more advanced maturation, there are several alternative explanations of findings. For example, given the angular resolution of the DTI sequence and known limitations in the tensor model, it cannot be ruled out that the data could be influenced by crossing fibers (Jones, Knösche, & Turner, 2013). When fibers cross, water molecules diffuse along the direction of each bundle, resulting in a ‘mixed’ diffusion signal and possibly lower FA and higher MD values. Such crossing may be indicative of typical development, hence in this case, it might be that children rejected and victimized had an atypical trajectory of white matter maturation involving less fiber crossing, resulting in higher FA and lower MD values. More advanced acquisition sequences and techniques would be required (Pasternak, Kelly, Sydnor, & Shenton, 2018) for spatial and angular resolution that can estimate measures of anisotropy and diffusivity that are less impacted by crossing fibers.

## Conclusion

In conclusion, we found that peer victimization and rejection were associated with higher FA and lower MD in white matter connections such as the corpus callosum and corona radiata. Children classified as ‘victims’ or ‘rejected’ had white matter microstructure differences equal to being 8 months older, yet repeated measurements are needed to unravel the temporal relations between peer connections, maturation and brain development. We highlight that results were largely consistent across white matter microstructure measurements (global and voxel‐wise, FA and MD), peer nomination scores (peer victimization and peer rejection), informants (self‐report vs. multi‐informant peer nominations) and statistical models (including different confounders, categorization of measures, adjusting for selection bias). This is the largest, most integrative study on social connections in childhood and white matter microstructure, indicating that peer connections in the classroom may be reflected in brain white matter connections.

## Supporting information


**Methods S1.** Image quality assurance.
**Methods S2**. Data imputation.
**Methods S3**. Tract‐based analyses.
**Methods S4**. Covariates.
**Methods S5**. Testing for influential outliers.
**Methods S6**. Inverse probability weighting.
**Methods S7**. Atlas labels.
**Results S1**. Influential outliers.
**Table S1**. Fiber tracts included in global diffusion tensor imaging metrics.
**Table S2**. Correlation matrix of variables of interest.
**Table S3**. Associations between PEERS measures and global white matter microstructure in base model.
**Table S4**. Associations of PEERS measures and covariates with global white matter microstructure in fully adjusted model.
**Table S5**. Associations of PEERS measures and covariates with global axial diffusivity (AD) and radial diffusivity (RD).
**Table S6**. Percentage of voxels per region for which peer victimization was associated with higher fractional anisotropy (FA) and lower mean diffusivity (MD) in base model (M1) and fully adjusted model (M2) using tract‐based spatial statistics.
**Table S7**. Percentage of voxels per region for which peer victimization was associated with lower radial diffusivity (RD).
**Table S8**. Tract‐based results for associations between peer victimization and fractional anisotropy (FA) and mean diffusivity (MD).
**Table S9**. Percentage of voxels per region for which peer rejection was associated with lower mean diffusivity (MD) in base model (M1) and fully adjusted model (M2) using tract‐based spatial statistics.
**Table S10**. Tract‐based results for associations between peer rejection and mean diffusivity (MD).
**Table S11**. Associations between peer victimization and global white matter microstructure– *outlier removed*.
**Table S12**. Inverse probability weighted associations between PEERS measures and global white matter microstructure.
**Table S13**. Associations between categorical measure of bullying‐involvement and global white matter microstructure.
**Table S14**. Percentage of voxels per region for which victims had higher fractional anisotropy (FA) and lower mean diffusivity (MD) than uninvolved children in categorized model using tract‐based spatial statistics.
**Table S15**. Associations between PEERS measures and global white matter microstructure ‐ with each PEERS measure in a separate model.
**Table S16**. Percentage of voxels per region for which peer victimization was associated with higher fractional anisotropy (FA) and lower mean diffusivity (MD)– unadjusted for bullying behavior.
**Figure S1**. Histograms of independent and dependent variables.
**Figure S2**. Histograms of residuals of analyses.
**Figure S3**. Scatter plots of nominally significant associations between PEERS measures and global white matter microstructure.
**Figure S4**. Scatter plots of nominally significant associations between peer victimization and global white matter microstructure – *outlier removed*.
