# Supplementary material for: Connecting the dots: social networks in the classroom and white matter connections in the brain
Source: J Child Psychol Psychiatry. 2022 Jun 7;63(12):1622–30. doi: 10.1111/jcpp.13647 (PMC9796140; doi:10.1111/jcpp.13647)
Supplement: Supplementary file 1 — Methods S1. Image quality assurance. Methods S2. Data imputation. Methods S3. Tract‐based analyses. Methods S4. Covariates. Methods S5. Testing for influential outliers. Methods S6. Inverse probability weighting. Methods S7. Atlas labels. Results S1. Influential outliers. Table S1. Fiber tracts included in global diffusion tensor imaging metrics. Table S2. Correlation matrix of variables of interest. Table S3. Associations between PEERS measures and global white matter microstructure in base model. Table S4. Associations of PEERS measures and covariates with global white matter microstructure in fully adjusted model. Table S5. Associations of PEERS measures and covariates with global axial diffusivity (AD) and radial diffusivity (RD). Table S6. Percentage of voxels per region for which peer victimization was associated with higher fractional anisotropy (FA) and lower mean diffusivity (MD) in base model (M1) and fully adjusted model (M2) using tract‐based spatial statistics. Table S7. Percentage of voxels per region for which peer victimization was associated with lower radial diffusivity (RD). Table S8. Tract‐based results for associations between peer victimization and fractional anisotropy (FA) and mean diffusivity (MD). Table S9. Percentage of voxels per region for which peer rejection was associated with lower mean diffusivity (MD) in base model (M1) and fully adjusted model (M2) using tract‐based spatial statistics. Table S10. Tract‐based results for associations between peer rejection and mean diffusivity (MD). Table S11. Associations between peer victimization and global white matter microstructure– outlier removed. Table S12. Inverse probability weighted associations between PEERS measures and global white matter microstructure. Table S13. Associations between categorical measure of bullying‐involvement and global white matter microstructure. Table S14. Percentage of voxels per region for which victims had higher fractional anisotropy (FA) and lower mean dif [file JCPP-63-1622-s001.docx]

**Supporting Information**

Connecting the dots: Social networks in the classroom and white matter connections in the brain

**Index**

*Supplemental Methods*

Methods S1. Image quality assurance

Methods S2. Data imputation

Methods S3. Tract-based analyses

Methods S4. Covariates

Methods S5. Testing for influential outliers

Methods S6. Inverse probability weighting

Methods S7. Atlas labels

*Supplemental Results*

Results S1. Influential outliers

*Supplemental Tables*

Table S1. Fiber tracts included in global diffusion tensor imaging metrics

Table S2. Correlation matrix of variables of interest

Table S3. Associations between PEERS measures and global white matter microstructure in base model

Table S4. Associations of PEERS measures and covariates with global white matter microstructure in fully adjusted model

Table S5. Associations of PEERS measures and covariates with global axial diffusivity (AD) and radial diffusivity (RD)

Table S6. Percentage of voxels per region for which peer victimization was associated with higher fractional anisotropy (FA) and lower mean diffusivity (MD) in base model (M1) and fully adjusted model (M2) using tract-based spatial statistics

Table S7. Percentage of voxels per region for which peer victimization was associated with lower radial diffusivity (RD)

Table S8. Tract-based results for associations between peer victimization and fractional anisotropy (FA) and mean diffusivity (MD)

Table S9. Percentage of voxels per region for which peer rejection was associated with lower mean diffusivity (MD) in base model (M1) and fully adjusted model (M2) using tract-based spatial statistics

Table S10. Tract-based results for associations between peer rejection and mean diffusivity (MD)

Table S11. Associations between peer victimization and global white matter microstructure– *outlier removed*

Table S12. Inverse probability weighted associations between PEERS measures and global white matter microstructure

Table S13. Associations between categorical measure of bullying-involvement and global white matter microstructure

Table S14. Percentage of voxels per region for which victims had higher fractional anisotropy (FA) and lower mean diffusivity (MD) than uninvolved children in categorized model using tract-based spatial statistics

Table S15. Associations between PEERS measures and global white matter microstructure - with each PEERS measure in a separate model

Table S16. Percentage of voxels per region for which peer victimization was associated with higher fractional anisotropy (FA) and lower mean diffusivity (MD)– unadjusted for bullying behavior

*Supplemental Figures*

Figure S1. Histograms of independent and dependent variables

Figure S2. Histograms of residuals of analyses

Figure S3. Scatter plots of nominally significant associations between PEERS measures and global white matter microstructure

Figure S4. Scatter plots of nominally significant associations between peer victimization and global white matter microstructure – *outlier removed*

**Supplemental Methods**

Methods S1. Image quality assurance

Several consecutive steps were taken to assure image quality of the diffusion-weighted data (*n*=3,992 for the full cohort of the Generation R Study; *n*=812 before quality control in the current study). First, cases with noticeable incidental findings, such as tumors or large cysts, were excluded from further analyses. Second, data of children who wore implants (e.g., retainers) which caused substantial artifact during the scanning procedure were excluded. Third, the diffusion-weighted data were automatically and manually assessed for quality (Muetzel et al., 2018). Each diffusion-weighted volume was automatically examined for slicewise variation, which is indicative of motion artifact, with the DTIPrep tool (<https://www.nitrc.org/projects/dtiprep/>). Cases with significant slicewise variation and flagged by DTIPrep as “Failed” were excluded. To adjust for any residual influences of slicewise variation, the number of volumes affected was added as a technical covariate (‘diffusion image quality’) to the models. In addition, voxel-wise sum-of-squares (SSE) maps from the diffusion-weighted data were visually inspected for structured signal indicative of artifact and excluded. Fourth, the quality of the processed tractography data was assessed by visual inspection of the registration to standard space and subsequent examination of the connectivity distribution of each tract for grossly misclassified voxels. After image quality assurance, diffusion-weighted data of 2,996 children were available for analyses. The data of 634 of these children were used in the current study.

*References*

Muetzel, R. L., Blanken, L. M. E., van der Ende, J., El Marroun, H., Shaw, P., Sudre, G., . . . Tiemeier, H. (2018). Tracking brain development and dimensional psychiatric symptoms in children: A longitudinal population-based neuroimaging study. Am. J. Psychiat., 175(1), 54-62.

Methods S2. Data imputation

Missing data on covariates (min n=0% for age and sex; max n=14% for household income) were imputed in R version 3.4.3 (R Core Team, 2013) using the mice package v3.13.0 (Buuren & Groothuis-Oudshoorn, 2010) with a maximum of 100 iterations creating 30 imputed datasets. For the analyses with the global metrics, estimates were pooled across the 30 imputed datasets. As the permutation software cannot account for missing covariates or accommodate imputed datasets, the TBSS analyses were performed on the 30th imputed dataset.

*References*

Buuren, S. v., & Groothuis-Oudshoorn, K. (2010). mice: Multivariate imputation by chained equations in R. Journal of statistical software, 1-68.

Methods S3. Tract-based analyses

To complement TBSS analyses, we performed follow-up analyses where we examined the association between the PEERS measure and tract-based DTI scalar metrics. These metrics were computed in native space by averaging DTI measures (e.g. FA) from all voxels within a given tract (Muetzel et al., 2018). These included the bilateral corticospinal tracts, bilateral superior longitudinal fasciculi, bilateral inferior longitudinal fasciculi, bilateral uncinated fasciculi, bilateral cingulate gurus part of the cingulum, and forceps minor and major of the corpus callosum (Table S1). Associations with *p*_FDR_<0.05 are reported.

*References*

Muetzel, R. L., Blanken, L. M. E., van der Ende, J., El Marroun, H., Shaw, P., Sudre, G., . . . Tiemeier, H. (2018). Tracking brain development and dimensional psychiatric symptoms in children: A longitudinal population-based neuroimaging study. Am. J. Psychiat., 175(1), 54-62.

Methods S4. Covariates

Child sex and date of birth were obtained from midwife and hospital records. The number of diffusion-weighted volumes that were automatically labelled by the DTIPrep tool (<https://www.nitrc.org/projects/dtiprep/>) to have signal attenuation indicative of motion artifact was used as a technical covariate (‘diffusion image quality’ Methods S1). Handedness was measured with an adaptation of the Edinburgh Handedness Inventory (Oldfield, 1971) at age 10 (ranging from -1 (all left) to 1 (all right)). Maternal education was used as an indicator of socio-economic status (university degree completed: yes or no). Parental national origin was measured as an indicator of parental ethnicity and immigration status and was determined by the parents’ country of birth (CBVD, 2004)(Dutch/Western, other than Dutch/non-Western). Net household monthly income was reported by the mother at child age 5 years and categorized into three groups (<2000 euros; 2000-3200 euros; >3200 euros). Non-verbal intelligence quotient (IQ) was measured with the Snijders-Oomen Non-verbal intelligence test – Revised (SON-R 2.5-7)(Tellegen, Winkel, & Wijnbergen-Williams, 1996) at 6 years. The IQ score was based on the subsets visuospatial abilities (Mosaics) and abstract reasoning (Categories) (*r*=0.15) and was normed based on the age of the child (Langeslag et al., 2013; Tellegen et al., 1996). Behavior problems were measured with the Child Behavior Checklist/6-18 (CBCL)(Achenbach & Rescorla, 2001) as reported by the primary caretaker at the age of 10 years. We selected the broadband scales Internalizing Problems (which includes items from the Anxious/Depressed, Withdrawn/Depressed, and Somatic Complaints syndrome scales) and Externalizing Problems (Rule-breaking Behavior and Aggressive Behavior) with 32 and 35 items, respectively, each rated on a 3-point scale. In the current sample, the internal reliability was α=0.81 for Internalizing Problems and α=0.84 for Externalizing Problems. Three additional variables were used for inverse probability weighting (see below): maternal age was assessed upon enrollment, gestational age at birth was determined using fetal ultrasound examinations, and birth weight was based on medical records.

*References*

Achenbach, T. M., & Rescorla, L. A. (2001). Manual for the ASEBA school-age forms & proﬁles. Burlington, VT: University of Vermont. Research Center for Children, Youth, & Families.

CBvd, Statistiek. "Immigrants in the Netherlands 2004." Netherland: Statistics Netherlands (2004).

Langeslag, S. J. E., Schmidt, M., Ghassabian, A., Jaddoe, V. W., Hofman, A., van der Lugt, A., . . . White, T. J. H. (2013). Functional connectivity between parietal and frontal brain regions and intelligence in young children: the Generation R study. Hum. Brain Mapp., 34(12), 3299-3307.

Oldfield, R. C. (1971). The assessment and analysis of handedness: the Edinburgh inventory. Neuropsychologia, 9(1), 97-113.

Tellegen, P. J., Winkel, M., & Wijnbergen-Williams, B. J. (1996). Snijders-Oomen Niet-verbale Intelligentietest-Revisie SON-R 2½–7: Lisse: Swets & Zeitlinger.

Methods S5. Testing for influential outliers

The PEERS measures were positively skewed (Figure S1), however transformation of the data did not result in normal distributions. Randomise is a non-parametric tool, thus the voxel-wise analyses are robust against non-normality of the data (Winkler, Ridgway, Webster, Smith, & Nichols, 2014). For the analyses of global white matter, we i) tested for non-normality of the residuals with a Kolmogorov-Smirnov test in each imputed set, ii) tested for influential data points using Cook’s distance in each imputed set, and iii) visually inspected scatter plots for outliers in the last imputed set. As a sensitivity analysis, any outlier(s) suspected to influence results were removed and analyses were repeated.

*References*

Winkler, A. M., Ridgway, G. R., Webster, M. A., Smith, S. M., & Nichols, T. E. (2014). Permutation inference for the general linear model. *Neuroimage*, *92*, 381-397.

Methods S6. Inverse probability weighting

Children included in the current study more often had parents that were born in the Netherlands, were older, and had mothers with higher education than children in the full Generation R Study (Kooijman et al., 2016). To adjust for potential selection effects, we used inverse probability weighting (Cole & Hernán, 2008) in a sensitivity analysis. Weights were computed on baseline characteristics child sex, parental national origin, education of the mother, age of the mother, and gestational age and weight at birth. Since “Randomise” does not allow for weighted analyses, we performed these analyses only for the global metrics.

*References*

Cole, S. R., & Hernán, M. A. (2008). Constructing inverse probability weights for marginal structural models. Am. J. Epidemiol., 168(6), 656-664.

Kooijman, M. N., Kruithof, C. J., van Duijn, C. M., Duijts, L., Franco, O. H., van IJzendoorn, M. H., . . . Mackenbach, J. P. (2016). The Generation R Study: design and cohort update 2017. European journal of epidemiology, 31(12), 1243-1264.

Methods S7. Atlas labels

To examine the localizations of the associated voxels in the tract-based spatial statistics (TBSS) analyses, voxels were labelled using the Johns Hopkins University (JHU) white matter tractography atlas (Mori, Van Zijl, & Nagae-Poetscher, 2005) and computed the percentage of associated voxels with regard to all available voxels in each tract. Since a number of voxels could not be labelled with the JHU atlas (or other available white matter atlases), we used the Harvard-Oxford cortical and subcortical atlas (Desikan et al., 2006) to confirm that the majority of these voxels were labelled as white matter. We note that these labels are merely meant to aid interpretation, since many voxels measured could not be labelled by the JHU atlas.

*References*

Desikan RS, Ségonne F, Fischl B, Quinn BT, Dickerson BC, Blacker D, Buckner RL, Dale AM, Maguire RP, Hyman BT, Albert MS, Killiany RJ. An automated labeling system for subdividing the human cerebral cortex on MRI scans into gyral based regions of interest. Neuroimage. 2006 Jul 1;31(3):968-80.

Mori, S., Wakana, S., Van Zijl, P. C., & Nagae-Poetscher, L. M. (2005). *MRI atlas of human white matter*. Elsevier.

**Supplemental Results**

Results S1. Influential outliers

Residuals of the global analyses showed no sign of non-normality (*p*>0.05, Figure S2) or influential data points (D_i_<1). However, after inspection of scatterplots for the results from the global we noted an outlier on peer victimization (Figure S3). After removal of the values for this participant, results remained consistent: a nominally significant association was found between peer victimization and global FA and MD in similar direction as the main results (Table S11; Figure S4).

**Supplemental Tables**

Table S1. Fiber tracts included in global diffusion tensor imaging metrics

|  |
| --- |
| *Projection fibers* |
| Corticospinal tract, left and right |
| *Association fibers* |
| Superior longitudinal fasciculus, left and right |
| Inferior longitudinal fasciculus, left and right |
| Uncinate fasciculus, left and right |
| *Limbic system fibers* |
| Cingulate gyrus part of cingulum, left and right |
| *Callosal fibers* |
| Forceps minor |
| Forceps major |

Table S2. Correlation matrix of variables of interest

|  | Bullying behavior | Peer rejection | Peer acceptance | Global FA | Global MD | Global AD | Global RD | Age at MRI | Diffusion image quality | Handedness | Non-verbal IQ | Internalizing problems | Externalizing problems |
| --- | --- | --- | --- | --- | --- | --- | --- | --- | --- | --- | --- | --- | --- |
| Peer victimization | 0.24* | 0.14* | 0.01 | 0.06 | -0.07 | -0.06 | -0.07 | 0.03 | 0.10* | 0.00 | -0.06 | 0.05 | 0.10* |
| Bullying behavior |  | 0.48* | -0.08* | -0.09* | 0.10* | 0.06 | 0.10* | 0.04 | 0.05 | -0.01 | -0.17* | 0.13* | 0.25* |
| Peer rejection |  |  | -0.36* | -0.01 | -0.06 | -0.06 | -0.04 | 0.06 | -0.03 | -0.01 | -0.14* | 0.01 | 0.15* |
| Peer acceptance |  |  |  | 0.02 | 0.00 | 0.00 | 0.00 | -0.04 | -0.02 | 0.01 | 0.03 | -0.08 | -0.13* |
| Global FA |  |  |  |  | -0.59* | 0.00 | -0.85* | 0.17* | -0.06 | 0.03 | 0.07 | -0.12* | -0.07 |
| Global MD |  |  |  |  |  | 0.76* | 0.92* | -0.14* | 0.04 | -0.05 | 0.02 | 0.03 | 0.00 |
| Global AD |  |  |  |  |  |  | 0.49* | -0.02 | 0.00 | -0.05 | 0.07 | -0.03 | -0.03 |
| Global RD |  |  |  |  |  |  |  | -0.18* | 0.05 | -0.05 | -0.02 | 0.07 | 0.02 |
| Age at MRI |  |  |  |  |  |  |  |  | -0.04 | 0.05 | -0.09* | -0.02 | 0.04 |
| Diffusion image quality |  |  |  |  |  |  |  |  |  | -0.07 | -0.02 | 0.04 | 0.06 |
| Handedness |  |  |  |  |  |  |  |  |  |  | -0.04 | 0.01 | -0.01 |
| Non-verbal IQ |  |  |  |  |  |  |  |  |  |  |  | -0.08 | -0.11* |
| Internalizing problems |  |  |  |  |  |  |  |  |  |  |  |  | 0.67* |

**p*<0.05

Table S3. Associations between PEERS measures and global white matter microstructure in base model

|  | Fractional anisotropy | | |  |  | Mean diffusivity | | | |  |
| --- | --- | --- | --- | --- | --- | --- | --- | --- | --- | --- |
|  | β | 95% CI | *p* | *p*_FDR_ | β | | 95% CI | *p* | *p*_FDR_ | |
| Peer victimization | 0.10 | 0.02; 0.18 | 0.02 | 0.04 | -0.09 | | -0.17; -0.01 | 0.02 | 0.04 | |
| Bullying behavior | -0.11 | -0.19; -0.03 | 0.01 | 0.04 | 0.06 | | -0.02; 0.14 | 0.12 | 0.20 | |
| Peer rejection | 0.00 | -0.08; 0.08 | 0.99 | 0.99 | -0.10 | | -0.18; -0.02 | 0.01 | 0.04 | |
| Peer acceptance | 0.02 | -0.06; 0.10 | 0.62 | 0.70 | -0.03 | | -0.11; 0.05 | 0.45 | 0.60 | |

*Note:* Results were adjusted for sex, age at MRI scan, diffusion image quality, handedness, and parental national origin.

*p* adj: *p*-value after

Table S4. Associations of PEERS measures and covariates with global white matter microstructure in fully adjusted model

|  | Fractional anisotropy | | |  | Mean diffusivity | | |
| --- | --- | --- | --- | --- | --- | --- | --- |
|  | β | 95% CI | *p* | β | | 95% CI | *p* |
| Peer victimization | 0.10 | 0.02; 0.18 | 0.01 | -0.09 | | -0.17; -0.01 | 0.03 |
| Bullying behavior | -0.08 | -0.17; 0.01 | 0.06 | 0.07 | | -0.02; 0.16 | 0.10 |
| Age at MRI | 0.27 | 0.14; 0.40 | 5.74x10^-05^ | 0.07 | | -0.01; 0.16 | 0.10 |
| Sex (boy) | 0.02 | -0.14; 0.18 | 0.80 | 0.02 | | -0.02; 0.06 | 0.34 |
| Diffusion image quality | -0.03 | -0.08; 0.01 | 0.12 | -0.26 | | -0.38; -0.13 | 9.21x10^-05^ |
| Ethnicity – Dutch vs non-Dutch, Western | -0.16 | -0.43; 0.10 | 0.23 | 0.43 | | 0.27; 0.59 | 2.01x10^-07^ |
| Ethnicity – Dutch vs non-Western | -0.19 | -0.39; 0.00 | 0.05 | 0.17 | | -0.09; 0.43 | 0.21 |
| Handedness | 0.04 | -0.12; 0.20 | 0.66 | -0.07 | | -0.24; 0.09 | 0.36 |
| Maternal education (university finished) | 0.21 | 0.02; 0.39 | 0.03 | -0.04 | | -0.22; 0.15 | 0.69 |
| Household income – low vs medium | 0.06 | -0.19; 0.31 | 0.65 | -0.15 | | -0.40; 0.10 | 0.23 |
| Household income – low vs high | 0.04 | -0.20; 0.28 | 0.75 | 0.02 | | -0.22; 0.27 | 0.86 |
| Non-verbal IQ | 0.00 | 0.00; 0.01 | 0.27 | 0.00 | | 0.00; 0.01 | 0.69 |
| Internalizing problems | 0.01 | -0.01; 0.03 | 0.50 | 0.00 | | -0.03; 0.02 | 0.71 |
| Externalizing problems | 0.00 | -0.02; 0.03 | 0.88 | -0.01 | | -0.03; 0.03 | 0.60 |
| Peer rejection | 0.02 | -0.07; 0.11 | 0.61 | -0.10 | | -0.19; -0.01 | 0.03 |
| Peer acceptance | 0.03 | -0.05; 0.11 | 0.42 | -0.04 | | -0.12; 0.04 | 0.39 |
| Age at MRI | 0.27 | 0.14; 0.40 | 4.91x10^-05^ | -0.26 | | -0.38; -0.13 | 9.87x10^-05^ |
| Sex (boy) | -0.03 | -0.19; 0.13 | 0.72 | 0.50 | | 0.34; 0.65 | 6.61x10^-10^ |
| Diffusion image quality | -0.03 | -0.07; 0.01 | 0.17 | 0.02 | | -0.03; 0.06 | 0.45 |
| Ethnicity – Dutch vs non-Dutch, Western | -0.14 | -0.41; 0.13 | 0.30 | 0.14 | | -0.13; 0.40 | 0.31 |
| Ethnicity – Dutch vs non-Western | -0.20 | -0.39; 0.00 | 0.05 | 0.05 | | -0.15; 0.24 | 0.63 |
| Handedness | 0.04 | -0.12; 0.20 | 0.64 | -0.08 | | -0.24; 0.13 | 0.56 |
| Maternal education (university finished) | 0.22 | 0.03; 0.41 | 0.02 | -0.05 | | -0.24; 0.13 | 0.56 |
| Household income – low vs medium | 0.07 | -0.18; 0.32 | 0.60 | -0.16 | | -0.41; 0.08 | 0.20 |
| Household income – low vs high | 0.05 | -0.19; 0.29 | 0.67 | -0.02 | | -0.26; 0.23 | 0.90 |
| Non-verbal IQ | 0.00 | 0.00; 0.01 | 0.21 | 0.00 | | -0.01; 0.01 | 0.92 |
| Internalizing problems | 0.01 | -0.01; 0.03 | 0.41 | -0.01 | | -0.03; 0.02 | 0.55 |
| Externalizing problems | 0.00 | -0.02; 0.02 | 0.99 | 0.00 | | -0.03; 0.02 | 0.84 |

Table S5. Associations of PEERS measures and covariates with global axial diffusivity (AD) and radial diffusivity (RD)

|  | Axial diffusivity | | |  | Radial diffusivity | | |  |
| --- | --- | --- | --- | --- | --- | --- | --- | --- |
|  | β | 95% CI | *p* | β | | 95% CI | *p* | |
| Peer victimization | -0.04 | -0.12; 0.04 | 0.26 | -0.10 | | -0.18; -0.02 | 0.01 | |
| Bullying behavior | 0.02 | -0.07; 0.11 | 0.56 | 0.08 | | -0.01; 0.17 | 0.08 | |
| Peer rejection | -0.10 | -0.18; -0.02 | 0.02 | -0.07 | | -0.16; 0.02 | 0.10 | |
| Peer acceptance | -0.03 | -0.11; 0.05 | 0.49 | -0.03 | | -0.11; 0.05 | 0.43 | |

*Note:* Results were adjusted for sex, age at MRI scan, diffusion image quality, handedness, parental national origin, non-verbal intelligence, maternal education, household income, internalizing and externalizing behavior problems.

Table S6. Percentage of voxels per region for which peer victimization was associated with higher fractional anisotropy (FA) and lower mean diffusivity (MD) in base model (M1) and fully adjusted model (M2)

| Percentage of voxels associated with respect to all voxels in region: | FA M1  (% voxels) | FA M2  (% voxels) | MD M1  (% voxels) | MD M2  (% voxels) |
| --- | --- | --- | --- | --- |
| JHU - Anterior corona radiata L | 0 | 0 | 79 | 57 |
| JHU - Anterior corona radiata R | 0 | 0 | 49 | 29 |
| JHU - Anterior limb of internal capsule L | 0 | 0 | 8 | 1 |
| JHU - Anterior limb of internal capsule R | 0 | 0 | 0 | 0 |
| JHU - Body of corpus callosum | 54 | 56 | 74 | 71 |
| JHU - Cerebral peduncle L | 0 | 0 | 0 | 0 |
| JHU - Cerebral peduncle R | 0 | 19 | 0 | 0 |
| JHU - Cingulum (cingulate gyrus) L | 2 | 2 | 0 | 0 |
| JHU - Cingulum (cingulate gyrus) R | 1 | 21 | 14 | 20 |
| JHU - Cingulum (hippocampus) L | 0 | 0 | 0 | 0 |
| JHU - Cingulum (hippocampus) R | 0 | 0 | 0 | 0 |
| JHU - Corticospinal tract L | 0 | 0 | 0 | 0 |
| JHU - Corticospinal tract R | 0 | 0 | 0 | 0 |
| JHU - External capsule L | 0 | 3 | 16 | 16 |
| JHU - External capsule R | 4 | 6 | 2 | 2 |
| JHU - Fornix (column and body of fornix) | 0 | 0 | 0 | 0 |
| JHU - Fornix (cres) / Stria terminalis (can not be resolved with current resolution) L | 6 | 14 | 14 | 13 |
| JHU - Fornix (cres) / Stria terminalis (can not be resolved with current resolution) R | 72 | 80 | 7 | 6 |
| JHU - Genu of corpus callosum | 0 | 0 | 31 | 28 |
| JHU - Inferior cerebellar peduncle L | 0 | 0 | 0 | 0 |
| JHU - Inferior cerebellar peduncle R | 0 | 0 | 0 | 0 |
| JHU - Medial lemniscus L | 0 | 0 | 0 | 0 |
| JHU - Medial lemniscus R | 0 | 0 | 0 | 0 |
| JHU - Middle cerebellar peduncle | 0 | 0 | 0 | 0 |
| JHU - Pontine crossing tract (a part of MCP) | 0 | 0 | 0 | 0 |
| JHU - Posterior corona radiata L | 56 | 53 | 56 | 56 |
| JHU - Posterior corona radiata R | 12 | 32 | 48 | 29 |
| JHU - Posterior limb of internal capsule L | 11 | 13 | 8 | 8 |
| JHU - Posterior limb of internal capsule R | 24 | 31 | 1 | 0 |
| JHU - Posterior thalamic radiation (include optic radiation) L | 0 | 0 | 63 | 61 |
| JHU - Posterior thalamic radiation (include optic radiation) R | 22 | 27 | 49 | 24 |
| JHU - Retrolenticular part of internal capsule L | 13 | 15 | 75 | 67 |
| JHU - Retrolenticular part of internal capsule R | 55 | 68 | 45 | 37 |
| JHU - Sagittal stratum (include inferior longitidinal fasciculus and inferior fronto-occipital fasciculus) L | 1 | 1 | 99 | 98 |
| JHU - Sagittal stratum (include inferior longitidinal fasciculus and inferior fronto-occipital fasciculus) R | 47 | 56 | 88 | 86 |
| JHU - Splenium of corpus callosum | 9 | 10 | 31 | 24 |
| JHU - Superior cerebellar peduncle L | 0 | 0 | 0 | 0 |
| JHU - Superior cerebellar peduncle R | 0 | 0 | 0 | 0 |
| JHU - Superior corona radiata L | 27 | 28 | 62 | 58 |
| JHU - Superior corona radiata R | 22 | 29 | 68 | 36 |
| JHU - Superior fronto-occipital fasciculus (could be a part of anterior internal capsule) L | 0 | 0 | 0 | 0 |
| JHU - Superior fronto-occipital fasciculus (could be a part of anterior internal capsule) R | 0 | 0 | 0 | 0 |
| JHU - Superior longitudinal fasciculus L | 5 | 4 | 72 | 71 |
| JHU - Superior longitudinal fasciculus R | 3 | 3 | 59 | 6 |
| JHU – Tapetum L | 0 | 0 | 0 | 0 |
| JHU - Tapetum R | 60 | 65 | 0 | 0 |
| JHU - Uncinate fasciculus L | 14 | 14 | 3 | 0 |
| JHU - Uncinate fasciculus R | 17 | 17 | 0 | 0 |
| Unclassified* | 5 | 5 | 35 | 27 |

FA: fractional anisotropy; MD: mean diffusivity; JHU: Johns Hopkins University white matter tractography atlas.

*95-96% of voxels unclassified by the JHU atlas were classified as white matter by the Harvard-Oxford atlas.

*Note:* base model M1 was adjusted for sex, age at MRI scan, diffusion image quality, handedness, and parental national origin. Fully adjusted M2 was additionally adjusted for non-verbal intelligence, maternal education, household income, internalizing and externalizing behavior problems.

Table S7. Percentage of voxels of voxels per region for which peer victimization was associated with lower radial diffusivity (RD)

| Percentage of voxels associated with respect to all voxels in region: | RD  (% voxels) |
| --- | --- |
| JHU - Anterior corona radiata L | 10 |
| JHU - Anterior corona radiata R | 23 |
| JHU - Anterior limb of internal capsule L | 0 |
| JHU - Anterior limb of internal capsule R | 37 |
| JHU - Body of corpus callosum | 84 |
| JHU - Cerebral peduncle L | 0 |
| JHU - Cerebral peduncle R | 21 |
| JHU - Cingulum (cingulate gyrus) L | 2 |
| JHU - Cingulum (cingulate gyrus) R | 23 |
| JHU - Cingulum (hippocampus) L | 0 |
| JHU - Cingulum (hippocampus) R | 0 |
| JHU - Corticospinal tract L | 0 |
| JHU - Corticospinal tract R | 0 |
| JHU - External capsule L | 13 |
| JHU - External capsule R | 7 |
| JHU - Fornix (column and body of fornix) | 0 |
| JHU - Fornix (cres) / Stria terminalis (can not be resolved with current resolution) L | 18 |
| JHU - Fornix (cres) / Stria terminalis (can not be resolved with current resolution) R | 84 |
| JHU - Genu of corpus callosum | 42 |
| JHU - Inferior cerebellar peduncle L | 0 |
| JHU - Inferior cerebellar peduncle R | 0 |
| JHU - Medial lemniscus L | 0 |
| JHU - Medial lemniscus R | 0 |
| JHU - Middle cerebellar peduncle | 0 |
| JHU - Pontine crossing tract (a part of MCP) | 0 |
| JHU - Posterior corona radiata L | 85 |
| JHU - Posterior corona radiata R | 66 |
| JHU - Posterior limb of internal capsule L | 16 |
| JHU - Posterior limb of internal capsule R | 44 |
| JHU - Posterior thalamic radiation (include optic radiation) L | 64 |
| JHU - Posterior thalamic radiation (include optic radiation) R | 61 |
| JHU - Retrolenticular part of internal capsule L | 67 |
| JHU - Retrolenticular part of internal capsule R | 87 |
| JHU - Sagittal stratum (include inferior longitidinal fasciculus and inferior fronto-occipital fasciculus) L | 68 |
| JHU - Sagittal stratum (include inferior longitidinal fasciculus and inferior fronto-occipital fasciculus) R | 89 |
| JHU - Splenium of corpus callosum | 58 |
| JHU - Superior cerebellar peduncle L | 0 |
| JHU - Superior cerebellar peduncle R | 0 |
| JHU - Superior corona radiata L | 50 |
| JHU - Superior corona radiata R | 89 |
| JHU - Superior fronto-occipital fasciculus (could be a part of anterior internal capsule) L | 0 |
| JHU - Superior fronto-occipital fasciculus (could be a part of anterior internal capsule) R | 50 |
| JHU - Superior longitudinal fasciculus L | 58 |
| JHU - Superior longitudinal fasciculus R | 73 |
| JHU - Tapetum L | 30 |
| JHU - Tapetum R | 82 |
| JHU - Uncinate fasciculus L | 14 |
| JHU - Uncinate fasciculus R | 17 |
| Unclassified* | 24 |

FA: fractional anisotropy; MD: mean diffusivity; JHU: Johns Hopkins University white matter tractography atlas.

*95% of voxels unclassified by the JHU atlas were classified as white matter by the Harvard-Oxford atlas.

*Note:* Results were adjusted for sex, age at MRI scan, diffusion image quality, handedness, parental national origin, non-verbal intelligence, maternal education, household income, internalizing and externalizing behavior problems.

Table S8. Tract-based results for associations between peer victimization and fractional anisotropy (FA) and mean diffusivity (MD)

|  | FA | | |  | MD | | |  |
| --- | --- | --- | --- | --- | --- | --- | --- | --- |
|  | β | 95% CI | *p* | *p*_FDR_ | β | 95% CI | *p* | *p*_FDR_ |
| *Projection fibers* |  |  |  |  |  |  |  |  |
| Corticospinal tract, left | 0.02 | -0.06; 0.10 | 0.66 | 0.90 | -0.02 | -0.10; 0.06 | 0.59 | 0.78 |
| Corticospinal tract, right | 0.02 | -0.06; 0.10 | 0.71 | 0.90 | 0.00 | -0.08; 0.08 | 0.93 | 0.93 |
| *Association fibers* |  |  |  |  |  |  |  |  |
| Superior longitudinal fasciculus, left | 0.12 | 0.04; 0.20 | 3.0x10^-03^ | 0.03 | -0.11 | -0.19; -0.04 | 4.0x10^-03^ | 0.05 |
| Superior longitudinal fasciculus, right | 0.11 | 0.03; 0.18 | 0.01 | 0.05 | -0.1 | -0.18; -0.02 | 0.01 | 0.06 |
| Inferior longitudinal fasciculus, left | 0.03 | -0.05; 0.11 | 0.42 | 0.72 | -0.08 | -0.15; 0.00 | 0.06 | 0.22 |
| Inferior longitudinal fasciculus, right | 0.07 | -0.01; 0.15 | 0.07 | 0.22 | -0.04 | -0.12; 0.03 | 0.28 | 0.55 |
| Uncinate fasciculus, left | 0.01 | -0.07; 0.09 | 0.90 | 0.90 | -0.01 | -0.09; 0.07 | 0.79 | 0.86 |
| Uncinate fasciculus, right | 0.03 | -0.05; 0.11 | 0.42 | 0.72 | -0.03 | -0.11; 0.05 | 0.45 | 0.68 |
| *Limbic system fibers* |  |  |  |  |  |  |  |  |
| Cingulate gyrus part of cingulum, left | 0.07 | -0.01; 0.14 | 0.11 | 0.25 | -0.07 | -0.15; 0.01 | 0.07 | 0.22 |
| Cingulate gyrus part of cingulum, right | 0.01 | -0.07; 0.09 | 0.81 | 0.90 | -0.01 | -0.09; 0.07 | 0.75 | 0.86 |
| *Callosal fibers* |  |  |  |  |  |  |  |  |
| Forceps minor | -0.01 | -0.09; 0.07 | 0.86 | 0.90 | -0.03 | -0.11; 0.05 | 0.45 | 0.68 |
| Forceps major | 0.08 | 0.00; 0.16 | 0.06 | 0.22 | -0.06 | -0.14; 0.02 | 0.11 | 0.27 |

*Note:* Results were adjusted for sex, age at MRI scan, diffusion image quality, handedness, parental national origin, non-verbal intelligence, maternal education, household income, internalizing and externalizing behavior problems.

*p*_FDR_ = false discovery rate corrected *p*-value over number of different tracts.

Table S9. Percentage of voxels per region for which peer rejection was associated with lower mean diffusivity (MD) in base model (M1) and fully adjusted model (M2) using tract-based spatial statistics

| Percentage of voxels associated with respect to all voxels in region: | MD M1  (% voxels) | | MD M2  (% voxels) |
| --- | --- | --- | --- |
| JHU - Anterior corona radiata L | | 12 | 0 |
| JHU - Anterior corona radiata R | | 70 | 0 |
| JHU - Anterior limb of internal capsule L | | 0 | 0 |
| JHU - Anterior limb of internal capsule R | | 3 | 0 |
| JHU - Body of corpus callosum | | 35 | 0 |
| JHU - Cerebral peduncle L | | 28 | 0 |
| JHU - Cerebral peduncle R | | 0 | 0 |
| JHU - Cingulum (cingulate gyrus) L | | 23 | 0 |
| JHU - Cingulum (cingulate gyrus) R | | 0 | 0 |
| JHU - Cingulum (hippocampus) L | | 0 | 0 |
| JHU - Cingulum (hippocampus) R | | 0 | 0 |
| JHU - Corticospinal tract L | | 0 | 0 |
| JHU - Corticospinal tract R | | 0 | 0 |
| JHU - External capsule L | | 5 | 0 |
| JHU - External capsule R | | 21 | 0 |
| JHU - Fornix (column and body of fornix) | | 0 | 0 |
| JHU - Fornix (cres) / Stria terminalis (can not be resolved with current resolution) L | | 72 | 0 |
| JHU - Fornix (cres) / Stria terminalis (can not be resolved with current resolution) R | | 0 | 0 |
| JHU - Genu of corpus callosum | | 58 | 0 |
| JHU - Inferior cerebellar peduncle L | | 0 | 0 |
| JHU - Inferior cerebellar peduncle R | | 0 | 0 |
| JHU - Medial lemniscus L | | 0 | 0 |
| JHU - Medial lemniscus R | | 0 | 0 |
| JHU - Middle cerebellar peduncle | | 0 | 0 |
| JHU - Pontine crossing tract (a part of MCP) | | 0 | 0 |
| JHU - Posterior corona radiata L | | 78 | 0 |
| JHU - Posterior corona radiata R | | 0 | 0 |
| JHU - Posterior limb of internal capsule L | | 68 | 0 |
| JHU - Posterior limb of internal capsule R | | 0 | 0 |
| JHU - Posterior thalamic radiation (include optic radiation) L | | 62 | 15 |
| JHU - Posterior thalamic radiation (include optic radiation) R | | 0 | 0 |
| JHU - Retrolenticular part of internal capsule L | | 89 | 2 |
| JHU - Retrolenticular part of internal capsule R | | 0 | 0 |
| JHU - Sagittal stratum (include inferior longitidinal fasciculus and inferior fronto-occipital fasciculus) L | | 77 | 14 |
| JHU - Sagittal stratum (include inferior longitidinal fasciculus and inferior fronto-occipital fasciculus) R | | 0 | 0 |
| JHU - Splenium of corpus callosum | | 53 | 0 |
| JHU - Superior cerebellar peduncle L | | 0 | 0 |
| JHU - Superior cerebellar peduncle R | | 0 | 0 |
| JHU - Superior corona radiata L | | 67 | 0 |
| JHU - Superior corona radiata R | | 15 | 0 |
| JHU - Superior fronto-occipital fasciculus (could be a part of anterior internal capsule) L | | 0 | 0 |
| JHU - Superior fronto-occipital fasciculus (could be a part of anterior internal capsule) R | | 0 | 0 |
| JHU - Superior longitudinal fasciculus L | | 47 | 21 |
| JHU - Superior longitudinal fasciculus R | | 0 | 0 |
| JHU - Tapetum L | | 55 | 0 |
| JHU - Tapetum R | | 0 | 0 |
| JHU - Uncinate fasciculus L | | 0 | 0 |
| JHU - Uncinate fasciculus R | | 56 | 0 |
| Unclassified* | | 23 | 1 |

FA: fractional anisotropy; MD: mean diffusivity; JHU: Johns Hopkins University white matter tractography atlas.

*92-100% of voxels unclassified by the JHU atlas were classified as white matter by the Harvard-Oxford atlas..

*Note:* Base model M1 was adjusted for sex, age at MRI scan, diffusion image quality, handedness, and parental national origin. Fully adjusted M2 was additionally adjusted for non-verbal intelligence, maternal education, household income, internalizing and externalizing behavior problems.

Table S10. Tract-based results for associations between peer rejection and mean diffusivity (MD)

|  | FA | | |  |
| --- | --- | --- | --- | --- |
|  | β | 95% CI | *p* | *p*_FDR_ |
| *Projection fibers* |  |  |  |  |
| Corticospinal tract, left | -0.07 | -0.16; 0.01 | 0.10 | 0.17 |
| Corticospinal tract, right | -0.10 | -0.19; -0.02 | 0.02 | 0.06 |
| *Association fibers* |  |  |  |  |
| Superior longitudinal fasciculus, left | -0.10 | -0.18; -0.02 | 0.02 | 0.06 |
| Superior longitudinal fasciculus, right | -0.06 | -0.14; 0.03 | 0.18 | 0.19 |
| Inferior longitudinal fasciculus, left | -0.08 | -0.16; 0.01 | 0.07 | 0.15 |
| Inferior longitudinal fasciculus, right | -0.06 | -0.14; 0.03 | 0.18 | 0.19 |
| Uncinate fasciculus, left | -0.10 | -0.18; -0.01 | 0.03 | 0.06 |
| Uncinate fasciculus, right | -0.10 | -0.19; -0.02 | 0.02 | 0.06 |
| *Limbic system fibers* |  |  |  |  |
| Cingulate gyrus part of cingulum, left | -0.07 | -0.15; 0.02 | 0.12 | 0.18 |
| Cingulate gyrus part of cingulum, right | -0.06 | -0.15; 0.02 | 0.15 | 0.19 |
| *Callosal fibers* |  |  |  |  |
| Forceps minor | -0.10 | -0.19; -0.01 | 0.02 | 0.06 |
| Forceps major | -0.04 | -0.13; 0.05 | 0.36 | 0.36 |

*Note:* Results were adjusted for sex, age at MRI scan, diffusion image quality, handedness, parental national origin, non-verbal intelligence, maternal education, household income, internalizing and externalizing behavior problems.

*p*_FDR_ = false discovery rate corrected *p*-value over number of different tracts.

Table S11. Associations between peer victimization and global white matter microstructure– *outlier removed*

|  | Fractional anisotropy | | |  | Mean diffusivity | | |  |
| --- | --- | --- | --- | --- | --- | --- | --- | --- |
|  | β | 95% CI | *p* | β | | 95% CI | *p* | |
| Peer victimization | 0.09 | 0.01; 0.17 | 0.03 | -0.08 | | -0.16; 0.00 | 0.04 | |
| Bullying behavior | -0.08 | -0.17; 0.01 | 0.07 | 0.07 | | -0.02; 0.16 | 0.10 | |

*Note:* Results were adjusted for sex, age at MRI scan, diffusion image quality, handedness, parental national origin, non-verbal intelligence, maternal education, household income, internalizing and externalizing behavior problems.

Table S12. Inverse probability weighted associations between PEERS measures and global white matter microstructure

|  | Fractional anisotropy | | |  | Mean diffusivity | | |  |
| --- | --- | --- | --- | --- | --- | --- | --- | --- |
|  | β | 95% CI | *p* | β | | 95% CI | *p* | |
| Peer victimization | 0.10 | 0.02; 0.18 | 0.02 | -0.08 | | -0.16; 0.00 | 0.04 | |
| Bullying behavior | -0.09 | -0.18; 0.00 | 0.04 | 0.07 | | -0.02; 0.16 | 0.12 | |
| Peer rejection | 0.02 | -0.07; 0.11 | 0.62 | -0.10 | | -0.19; -0.01 | 0.02 | |
| Peer acceptance | 0.04 | -0.04; 0.12 | 0.39 | -0.04 | | -0.12; 0.04 | 0.33 | |

*Note:* Results were adjusted for sex, age at MRI scan, diffusion image quality, handedness, parental national origin, non-verbal intelligence, maternal education, household income, internalizing and externalizing behavior problems.

Table S13. Associations between categorical measure of bullying-involvement and global white matter microstructure

|  | Global fractional anisotropy | | | Global mean diffusivity | | |
| --- | --- | --- | --- | --- | --- | --- |
|  | β | 95% CI | *p* | β | 95% CI | *p* |
| Victim vs uninvolved | 0.18 | -0.04; 0.40 | 0.10 | -0.18 | -0.40; 0.04 | 0.11 |
| Bully vs uninvolved | -0.12 | -0.37; 0.13 | 0.35 | 0.01 | -0.24; 0.26 | 0.91 |
| Bully-victim vs uninvolved | -0.06 | -0.42; 0.30 | 0.73 | -0.05 | -0.40; 0.30 | 0.77 |
| Age | 0.27 | 0.14; 0.40 | 4.12x10^-05^ | -0.26 | -0.39; -0.13 | 7.95x10^-05^ |
| Sex (boy) | 0.00 | -0.16; 0.16 | 0.99 | 0.46 | -0.30; 0.62 | 1.65x10^-08^ |
| Diffusion image quality | -0.03 | -0.07; 0.01 | 0.18 | 0.02 | -0.02; 0.06 | 0.40 |
| Ethnicity – Dutch vs non-Dutch, Western | -0.13 | -0.40; 0.13 | 0.33 | 0.15 | -0.12; 0.41 | 0.28 |
| Ethnicity – Dutch vs non-Western | -0.20 | -0.40; 0.00 | 0.05 | 0.05 | -0.15; 0.24 | 0.62 |
| Handedness | 0.04 | -0.12; 0.20 | 0.65 | -0.08 | -0.24; 0.09 | 0.36 |
| Maternal education (university finished) | 0.21 | 0.02; 0.40 | 0.03 | -0.04 | -0.23; 0.14 | 0.64 |
| Household income – low vs medium | 0.04 | -0.21; 0.29 | 0.74 | -0.15 | -0.40; 0.10 | 0.24 |
| Household income – low vs high | 0.02 | -0.22; 0.26 | 0.85 | 0.02 | -0.22; 0.27 | 0.84 |
| Non-verbal IQ | 0.00 | 0.00; 0.01 | 0.24 | 0.00 | -0.01; 0.01 | 0.80 |
| Internalizing problems | 0.01 | -0.01; 0.03 | 0.45 | -0.01 | -0.03; 0.02 | 0.61 |
| Externalizing problems | 0.00 | -0.02; 0.02 | 0.97 | 0.00 | -0.03; 0.02 | 0.76 |

Table S14. Percentage of voxels per region for which victims had higher fractional anisotropy (FA) and lower mean diffusivity (MD) than uninvolved children in categorized model

| Percentage of voxels associated with respect to all voxels in region: | FA  (% voxels) | MD  (% voxels) |
| --- | --- | --- |
| JHU - Anterior corona radiata L | 0 | 4 |
| JHU - Anterior corona radiata R | 0 | 4 |
| JHU - Anterior limb of internal capsule L | 0 | 0 |
| JHU - Anterior limb of internal capsule R | 0 | 0 |
| JHU - Body of corpus callosum | 42 | 30 |
| JHU - Cerebral peduncle L | 0 | 0 |
| JHU - Cerebral peduncle R | 30 | 0 |
| JHU - Cingulum (cingulate gyrus) L | 2 | 0 |
| JHU - Cingulum (cingulate gyrus) R | 3 | 0 |
| JHU - Cingulum (hippocampus) L | 0 | 0 |
| JHU - Cingulum (hippocampus) R | 0 | 0 |
| JHU - Corticospinal tract L | 0 | 0 |
| JHU - Corticospinal tract R | 0 | 0 |
| JHU - External capsule L | 0 | 2 |
| JHU - External capsule R | 0 | 0 |
| JHU - Fornix (column and body of fornix) | 0 | 0 |
| JHU - Fornix (cres) / Stria terminalis (can not be resolved with current resolution) L | 0 | 6 |
| JHU - Fornix (cres) / Stria terminalis (can not be resolved with current resolution) R | 0 | 0 |
| JHU - Genu of corpus callosum | 22 | 0 |
| JHU - Inferior cerebellar peduncle L | 0 | 0 |
| JHU - Inferior cerebellar peduncle R | 0 | 0 |
| JHU - Medial lemniscus L | 0 | 0 |
| JHU - Medial lemniscus R | 0 | 0 |
| JHU - Middle cerebellar peduncle | 0 | 0 |
| JHU - Pontine crossing tract (a part of MCP) | 0 | 0 |
| JHU - Posterior corona radiata L | 44 | 21 |
| JHU - Posterior corona radiata R | 9 | 14 |
| JHU - Posterior limb of internal capsule L | 0 | 0 |
| JHU - Posterior limb of internal capsule R | 40 | 0 |
| JHU - Posterior thalamic radiation (include optic radiation) L | 0 | 6 |
| JHU - Posterior thalamic radiation (include optic radiation) R | 6 | 0 |
| JHU - Retrolenticular part of internal capsule L | 0 | 37 |
| JHU - Retrolenticular part of internal capsule R | 56 | 0 |
| JHU - Sagittal stratum (include inferior longitidinal fasciculus and inferior fronto-occipital fasciculus) L | 0 | 80 |
| JHU - Sagittal stratum (include inferior longitidinal fasciculus and inferior fronto-occipital fasciculus) R | 0 | 29 |
| JHU - Splenium of corpus callosum | 0 | 0 |
| JHU - Superior cerebellar peduncle L | 0 | 0 |
| JHU - Superior cerebellar peduncle R | 0 | 0 |
| JHU - Superior corona radiata L | 22 | 42 |
| JHU - Superior corona radiata R | 5 | 26 |
| JHU - Superior fronto-occipital fasciculus (could be a part of anterior internal capsule) L | 0 | 0 |
| JHU - Superior fronto-occipital fasciculus (could be a part of anterior internal capsule) R | 0 | 0 |
| JHU - Superior longitudinal fasciculus L | 3 | 2 |
| JHU - Superior longitudinal fasciculus R | 0 | 1 |
| JHU - Tapetum L | 0 | 0 |
| JHU - Tapetum R | 12 | 0 |
| JHU - Uncinate fasciculus L | 0 | 0 |
| JHU - Uncinate fasciculus R | 0 | 0 |
| Unclassified* | 2 | 8 |

FA: fractional anisotropy; MD: mean diffusivity; JHU: Johns Hopkins University white matter tractography atlas.

*95-98% of voxels unclassified by the JHU atlas were classified as white matter by the Harvard-Oxford atlas.

*Note:* Results were adjusted for sex, age at MRI scan, diffusion image quality, handedness, parental national origin, non-verbal intelligence, maternal education, household income, internalizing and externalizing behavior problems

Table S15. Associations between PEERS measures and global white matter - with each PEERS measure in a separate model

|  | Fractional anisotropy | | |  | Mean diffusivity | | |  |
| --- | --- | --- | --- | --- | --- | --- | --- | --- |
|  | β | 95% CI | *p* | β | | 95% CI | *p* | |
| Peer victimization | 0.09 | 0.01; 0.17 | 0.03 | -0.08 | | -0.16; 0.00 | 0.05 | |
| Bullying behavior | -0.06 | -0.15; 0.03 | 0.17 | 0.05 | | -0.03; 0.13 | 0.23 | |
| Peer rejection | 0.01 | -0.07; 0.09 | 0.80 | -0.09 | | -0.17; -0.01 | 0.04 | |
| Peer acceptance | 0.03 | -0.05; 0.11 | 0.50 | 0.00 | | -0.08; 0.08 | 0.92 | |

*Note:* Results were adjusted for sex, age at MRI scan, diffusion image quality, handedness, parental national origin, non-verbal intelligence, maternal education, household income, internalizing and externalizing behavior problems.

Table S16. Percentage of voxels per region for which peer victimization was associated with higher fractional anisotropy (FA) and lower mean diffusivity (MD)– unadjusted for bullying behavior

| Percentage of voxels associated with respect to all voxels in region: | FA  (% voxels) | MD (% voxels) |
| --- | --- | --- |
| JHU - Anterior corona radiata L | 0 | 12 |
| JHU - Anterior corona radiata R | 0 | 28 |
| JHU - Anterior limb of internal capsule L | 0 | 0 |
| JHU - Anterior limb of internal capsule R | 0 | 0 |
| JHU - Body of corpus callosum | 7 | 63 |
| JHU - Cerebral peduncle L | 0 | 0 |
| JHU - Cerebral peduncle R | 0 | 0 |
| JHU - Cingulum (cingulate gyrus) L | 0 | 0 |
| JHU - Cingulum (cingulate gyrus) R | 0 | 6 |
| JHU - Cingulum (hippocampus) L | 0 | 0 |
| JHU - Cingulum (hippocampus) R | 0 | 0 |
| JHU - Corticospinal tract L | 0 | 0 |
| JHU - Corticospinal tract R | 0 | 0 |
| JHU - External capsule L | 0 | 4 |
| JHU - External capsule R | 0 | 2 |
| JHU - Fornix (column and body of fornix) | 0 | 0 |
| JHU - Fornix (cres) / Stria terminalis (can not be resolved with current resolution) L | 0 | 7 |
| JHU - Fornix (cres) / Stria terminalis (can not be resolved with current resolution) R | 0 | 0 |
| JHU - Genu of corpus callosum | 0 | 11 |
| JHU - Inferior cerebellar peduncle L | 0 | 0 |
| JHU - Inferior cerebellar peduncle R | 0 | 0 |
| JHU - Medial lemniscus L | 0 | 0 |
| JHU - Medial lemniscus R | 0 | 0 |
| JHU - Middle cerebellar peduncle | 0 | 0 |
| JHU - Pontine crossing tract (a part of MCP) | 0 | 0 |
| JHU - Posterior corona radiata L | 0 | 46 |
| JHU - Posterior corona radiata R | 0 | 29 |
| JHU - Posterior limb of internal capsule L | 0 | 8 |
| JHU - Posterior limb of internal capsule R | 0 | 0 |
| JHU - Posterior thalamic radiation (include optic radiation) L | 0 | 50 |
| JHU - Posterior thalamic radiation (include optic radiation) R | 0 | 7 |
| JHU - Retrolenticular part of internal capsule L | 0 | 67 |
| JHU - Retrolenticular part of internal capsule R | 0 | 21 |
| JHU - Sagittal stratum (include inferior longitidinal fasciculus and inferior fronto-occipital fasciculus) L | 0 | 82 |
| JHU - Sagittal stratum (include inferior longitidinal fasciculus and inferior fronto-occipital fasciculus) R | 0 | 72 |
| JHU - Splenium of corpus callosum | 0 | 2 |
| JHU - Superior cerebellar peduncle L | 0 | 0 |
| JHU - Superior cerebellar peduncle R | 0 | 0 |
| JHU - Superior corona radiata L | 14 | 46 |
| JHU - Superior corona radiata R | 0 | 32 |
| JHU - Superior fronto-occipital fasciculus (could be a part of anterior internal capsule) L | 0 | 0 |
| JHU - Superior fronto-occipital fasciculus (could be a part of anterior internal capsule) R | 0 | 0 |
| JHU - Superior longitudinal fasciculus L | 0 | 69 |
| JHU - Superior longitudinal fasciculus R | 0 | 3 |
| JHU - Tapetum L | 0 | 0 |
| JHU - Tapetum R | 0 | 0 |
| JHU - Uncinate fasciculus L | 0 | 0 |
| JHU - Uncinate fasciculus R | 0 | 0 |
| Unclassified* | 0 | 17 |

FA: fractional anisotropy; MD: mean diffusivity; JHU: Johns Hopkins University white matter tractography atlas.

*95-100% of voxels unclassified by the JHU atlas were classified as white matter by the Harvard-Oxford atlas.

*Note:* Results were adjusted for sex, age at MRI scan, diffusion image quality, handedness, parental national origin, non-verbal intelligence, maternal education, household income, internalizing and externalizing behavior problems.

**Supplemental Figures**

**
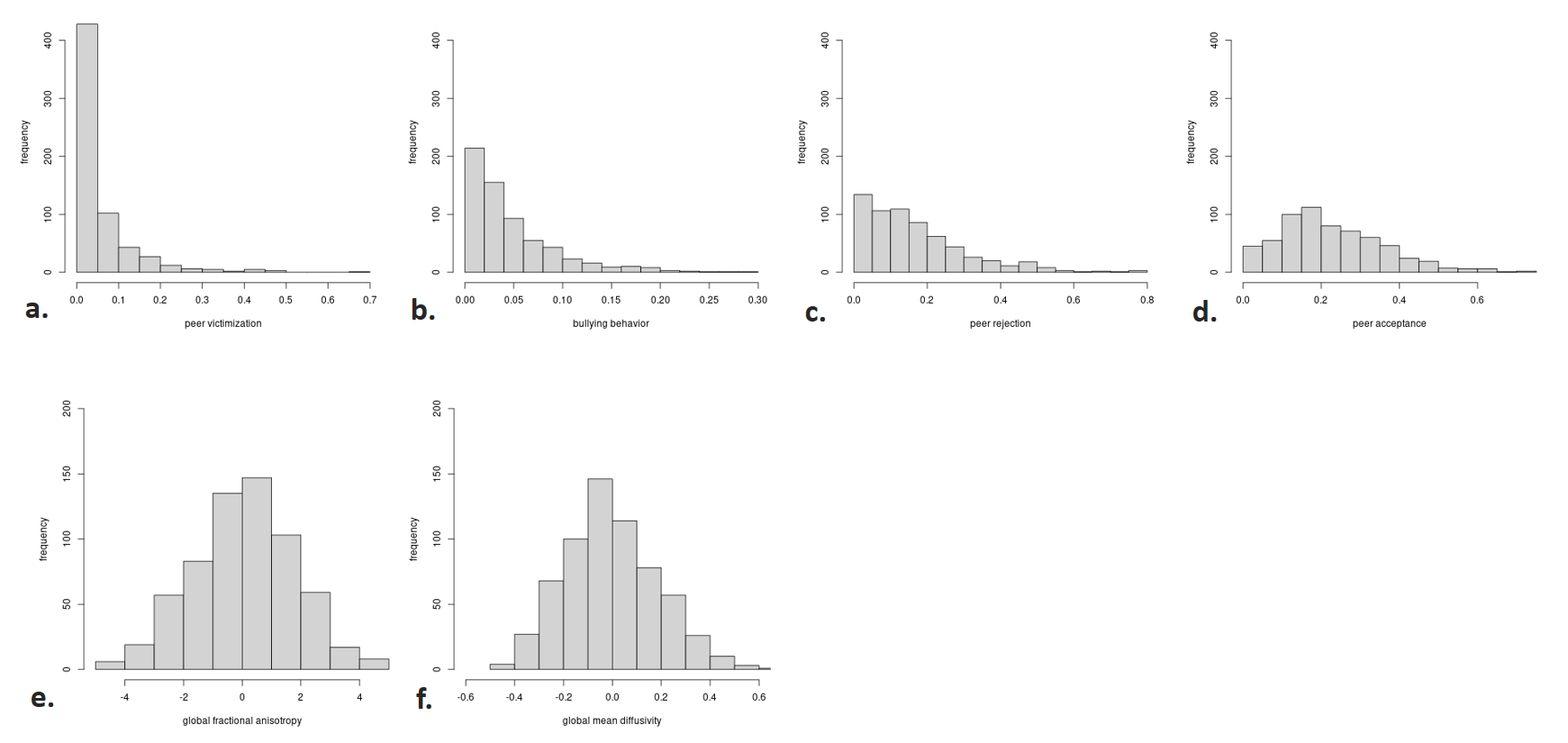
**

Figure S1. Histograms of independent and dependent variables, including a) peer victimization, b) bullying behavior, c) peer rejection, d) peer acceptance, e) fractional anisotropy, and f) mean diffusivity.


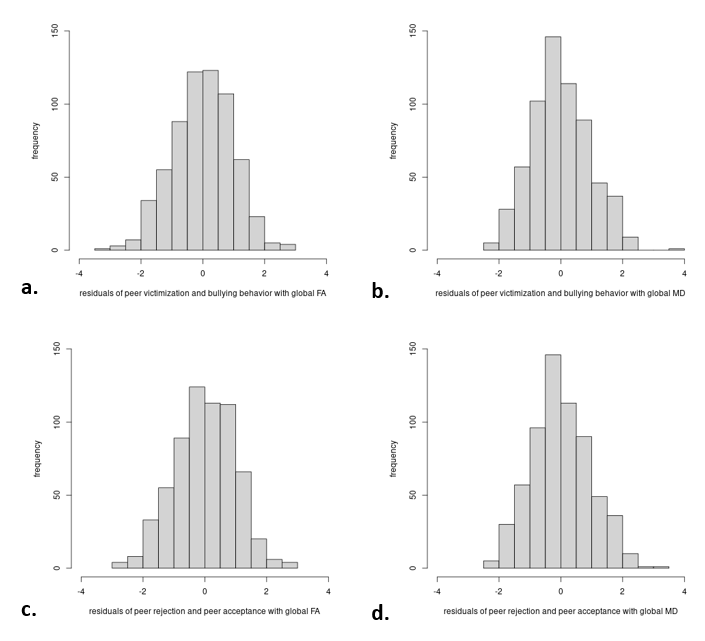


Figure S2. Histograms of residuals of analyses of a) peer victimization and bullying behaviour and global FA, b) peer victimization and bullying behaviour and global MD, c) peer rejection and peer acceptance and global MD, d) peer rejection and peer acceptance and global MD. Analyses were adjusted for sex, age at MRI scan, diffusion image quality, handedness, parental national origin, non-verbal intelligence, maternal education, household income, internalizing and externalizing behavior problems. Residuals portrayed are from the last imputed set.


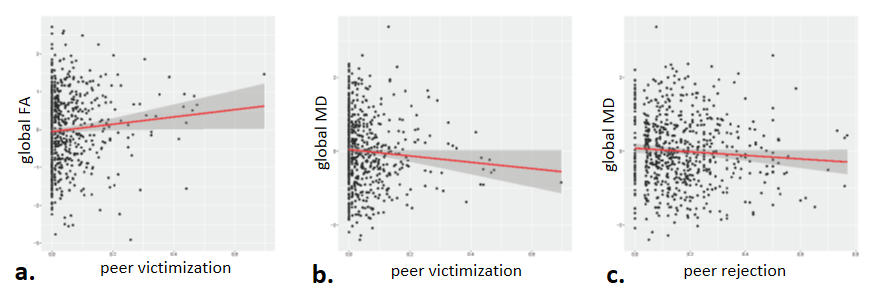


Figure S3. Scatter plots of nominally significant associations between PEERS measures and global white matter microstructure. Red line with 95% confidence level indicates linear association between peer victimization and global white matter structure, residualized for sex, age at MRI scan, diffusion image quality, handedness, parental national origin, non-verbal intelligence, maternal education, household income, internalizing and externalizing behavior problems.


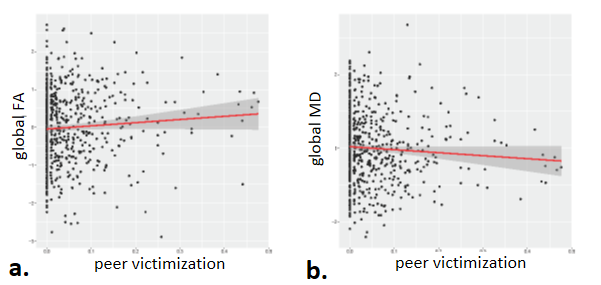


Figure S4. Scatter plots of nominally significant associations between peer victimization and global white matter microstructure – *outlier removed.* Red line with 95% confidence level indicates linear association between peer victimization and global white matter structure, residualized for sex, age at MRI scan, diffusion image quality, handedness, parental national origin, non-verbal intelligence, maternal education, household income, internalizing and externalizing behavior problems.
